# Telemedicine and urban diabetes during COVID-19 pandemic in Milano, Italy during lock-down: epidemiological and sociodemographic picture

**DOI:** 10.1007/s00592-021-01700-2

**Published:** 2021-03-19

**Authors:** Livio Luzi, Michele Carruba, Roberta Crialesi, Stefano Da Empoli, Regina Dagani, Elisabetta Lovati, Antonio Nicolucci, Cesare C. Berra, Elisa Cipponeri, Ketty Vaccaro, Andrea Lenzi

**Affiliations:** 1 Department of Biomedical Sciences for Health, University of Milan, Milan, Italy; 2 Department of Endocrinology, Nutrition and Metabolic Diseases, IRCCS Multimedica, Via Milanese 300, 20099 Sesto San Giovanni, Milan, Italy; 3 Department of Medical Biotechnology and Translational Medicine (BIOMETRA), University of Milan, Milan, Italy; 4 Centre for Study and Research on Obesity of the University of Milan, Milan, Italy; 5 National Institute of Statistics of Italy - ISTAT, Rome, Italy; 6 Institute for Competitiveness - I-COM, Rome, Italy; 7 Italian Diabetes Society Foundation Association – AMD Lombardy, Milan, Italy; 8 Health Agencies in the Territories (ASST) Rhodense, Milan, Italy; 9 Health City Institute, Rome, Italy; 10 Department Experimental Medicine, La Sapienza University, Rome, Italy; 11 Biotechnology and Life Sciences of Prime Minister Council – CNBBSV, Rome, Italy; 12 Italian Diabetes Society – SID Lombardy, Pavia, Italy; 13 I.R.C.C.S. Policlinico San Matteo, Pavia, Italy; 14 Centre for Outcomes Research and Clinical Epidemiology - CORESEARCH, Pescara, Italy; 15 Fondazione CENSIS, Rome, Italy

**Keywords:** Urban diabetes, Diabetes and obesity prevalence, COVID-19, Telemonitoring of blood glucose

## Abstract

**Background:**

Since 2010, more than half of World population lives in Urban Environments. Urban Diabetes has arisen as a novel nosological entity in Medicine. Urbanization leads to the accrual of a number of factors increasing the vulnerability to diabetes mellitus and related diseases. Herein we report clinical-epidemiological data of the Milano Metropolitan Area in the contest of the Cities Changing Diabetes Program. Since the epidemiological picture was taken in January 2020, on the edge of COVID-19 outbreak in the Milano Metropolitan Area, a perspective addressing potential interactions between diabetes and obesity prevalence and COVID-19 outbreak, morbidity and mortality will be presented. To counteract lock-down isolation and, in general, social distancing a pilot study was conducted to assess the feasibility and efficacy of tele-monitoring via Flash Glucose control in a cohort of diabetic patients in ASST North Milano.

**Methods:**

Data presented derive from 1. ISTAT (National Institute of Statistics of Italy), 2. Milano ATS web site (Health Agency of Metropolitan Milano Area), which entails five ASST (Health Agencies in the Territories). A pilot study was conducted in 65 screened diabetic patients (only 40 were enrolled in the study of those 36 were affected by type 2 diabetes and 4 were affected by type 1 diabetes) of ASST North Milano utilizing Flash Glucose Monitoring for 3 months (mean age 65 years, HbA1c 7,9%. Patients were subdivided in 3 groups using glycemic Variability Coefficient (VC): a. High risk, VC > 36, n. 8 patients; Intermediate risk 20 < VC < 36, n. 26 patients; Low risk VC < 20, n. 4 patients. The control group was constituted by 26 diabetic patients non utilizing Flash Glucose monitoring.

**Results:**

In a total population of 3.227.264 (23% is over 65 y) there is an overall prevalence of 5.65% with a significant difference between Downtown ASST (5.31%) and peripheral ASST (ASST North Milano, 6.8%). Obesity and overweight account for a prevalence of 7.8% and 27.7%, respectively, in Milano Metropolitan Area. We found a linear relationship (*R* = 0.36) between prevalence of diabetes and aging index. Similarly, correlations between diabetes prevalence and both older people depending index and structural dependence index (*R* = 0.75 and *R* = 0.93, respectively), were found. A positive correlation (*R* = 0.46) with percent of unoccupied people and diabetes prevalence was also found. A reverse relationship between diabetes prevalence and University level instruction rate was finally identified (*R* = − 0.82). Our preliminary study demonstrated a reduction of Glycated Hemoglobin (*p* = 0.047) at 3 months follow-up during the lock-down period, indicating Flash Glucose Monitoring and remote control as a potential methodology for diabetes management during COVID-19 lock-down.

**Hypothesis and discussion:**

The increase in diabetes and obesity prevalence in Milano Metropolitan Area, which took place over 30 years, is related to several environmental factors. We hypothesize that some of those factors may have also determined the high incidence and virulence of COVID-19 in the Milano area. Health Agencies of Milano Metropolitan Area are presently taking care of diabetic patients facing the new challenge of maintaining sustainable diabetes care costs in light of an increase in urban population and of the new life-style. The COVID-19 pandemic will modify the management of diabetic and obese patients permanently, via the implementation of approaches that entail telemedicine technology. The pilot study conducted during the lock-down period indicates an improvement of glucose control utilizing a remote glucose control system in the Milano Metropolitan Area, suggesting a wider utilization of similar methodologies during the present “second wave” lock-down.

## Introduction

Diabetes prevalence is increasing at an alarming pace World-wide. The World prevalence of the disease almost doubled in the last 16 years, increasing from 4.6% in 2000 [1] up to 9.1% in 2017 [2]. It has been estimated that diabetes prevalence will reach 11.7% (736 Millions people) in 2045, unless specific actions are taken. No Health System World-wide would be able to sustain this growth.

Along with diabetes mellitus, aging of the population and most chronic-degenerative diseases like obesity, hypertension, Chronic-Obstructive Pulmonary Disease (COPD) are increased in the urban environment. This is mainly due to environmental factors specific of cities with respect to rural areas. Sedentariness, pollution and closed environments may favor the spread of diabetes mellitus and the related metabolic syndrome [2]. Nowadays, 3.9 Billion people (over 50% of World population) live inside the cities, and it is estimated that the figure will reach 6.3 Billion people in 2050 [3]. Thus, Cities will become more and more the frontier to fight the rise of Type 2 diabetes mellitus, obesity and the other clinical conditions causing the metabolic syndrome. In 2014 University College of London, Steno Diabetes Centre of Copenhagen and Novo-Nordisk started a global program—the Cities Changing Diabetes to implement the global fight against diabetes [4]. Up today, the program includes 26 Megalopolis world-wide to face social, cultural and environmental determinants potentially increasing the diabetes vulnerability of people living in urban areas. The Milano Metropolitan Area entered the program in 2018. The initial phase consisted of mapping the clinical-epidemiology and sociodemographic landscape of Urban Diabetes in Milano up to January 2019.

On January 31, 2020 the Italian Government declared a national Emergency due to COVID-19 pandemic, which is still in due course. The COVID-19 pandemic began in a large city, Wuhan (Hubei province, China), during the month of December 2019 and is spreading around the World hitting mainly large Metropolis (e.g., Paris, London, Moscow, New York City) including Milano [5]. The viral pathogen belongs to the family of *Coronaviridae*, and is similar to SARS-CoV that determined an outbreak in 2003, mainly in China and in the Americas [6]. Very likely SARS-CoV-2 derives from a *casual* mutation in a bat host, capable of spreading inside the human host [7]. The modality of COVID-19 spreading mainly throughout cities is still *casual*? Or, in contrast, the urban environment is *necessary* for the diffusion of the virus around the World population? Diabetes and obesity, as well as hypertension and COPD are co-morbidities that may facilitate contagion with SARS-CoV-2 and worsen the prognosis of COVID-19 [8, 9]. Therefore, urban districts with high population density and high prevalence of diabetes and obesity may contribute to viral spreading and virulence.

The present article is composed by 2 parts: 1. A perspective linking the Clinical-Epidemiological map of the Milano Metropolitan Area in the different Health Sanitary Territories with COVID-19 pandemic and Urban Environment, with a specific focus on the role playable by diabetes mellitus, obesity and environmental factors of Urban settings; 2. A pilot study showing a potential role of remote glucose control and telemedicine in improving glucose control in patients with diabetes during lock-down periods.

## Methods

1. National Institute of Statistics (ISTAT) provided social and demographic data presented [10]. Diabetes prevalence for each Municipality belonging to the Milano Metropolitan Area was derived from data on health condition of the population made public by the Department of Epidemiology of the Agency for Health Maintenance of Milano Metropolitan Area [11]. Prevalence of Obesity and sedentariness was obtained from National Surveys of National Institute of Statistics (ISTAT) [12]. Unemployment rates were derived from a report of Ministry of Economy and Finance and National Institute of Statistics [13]. National Institute of Statistics was the source of data on Universities registration records [14, 15].
  Parameters calculated: 1. Aging Index (Number of Inhabitants over 65/Number of Children 0–14 y); 2. Old Age dependency ratio (Number of Inhabitants over 65y/Active Inhabitants 15–64 y); Structural Dependency ratio [Inactive Inhabitants (0–14 y and over 65 y)/(Active Inhabitants 15–64 y); unemployment rate (percent of Inhabitants between 15 and 64 y without occupation) and University Index (Inhabitants over 15 y registered to Universities and total Inhabitants over 15 y).
2. The present case control study was designed to test whether remote continuity of care using the LIBREVIEW° platform influenced glycaemic variability and metabolic control in people with diabetes on insulin therapy during pandemic compared to people with diabetes, on insulin therapy and not using LIBREVIEW° platform (control group).

Cases were identified among adult people (> 18 years old) both type 1 [4] and type 2 diabetes [16] already in possession of Flash Glucose Monitoring Device (FGM), from at least 1 year, and on insulin therapy. Controls matched for age and sex with cases were identified among patients with type 1 and type 2 diabetes. Both cases and controls were referring to the Department of Endocrinology, Nutrition and Metabolic Disease of Multimedica Hospital Group in Milan.

Clinical data concerning control were retrieved backward through computerized clinical records. T0 data were collected from visits made at outpatient clinic between January 2 and February 26, while T1 data were collected from visits made from June up to September.

Cases were contacted by phone from February 26 up to May 15. Patients were subdivided in 3 groups using glycemic Variability Coefficient (VC): a. High risk, VC > 36, n. 8 patients; Intermediate risk 20 < VC < 36, n. 26 patients; Low risk VC < 20, n. 4 patients. Assessment of glycemic compensation was performed weekly for the first group, twice a week for the second and monthly for the low-risk group. During the follow-up period (3 months) the following parameters were evaluated: hypoglycemic and hyperglycemic events and the standard deviation of glycemic excursion Body weight, height, glycated hemoglobin (HbA1c) and body max index (BMI) calculated as weight in kilograms divided by the square of height in meters, were recorded both for case and control groups.

### Statistical analysis

Linear regression analysis and Pearson coefficient of correlation were utilized to assess the relationship between diabetes prevalence and single social-demographic-economic indicators.

Statistical differences between the group wearing the device for remote glucose control and the control group were determined with ANOVA and unpaired t-test when appropriate.

## Results

**Fig. 1:**
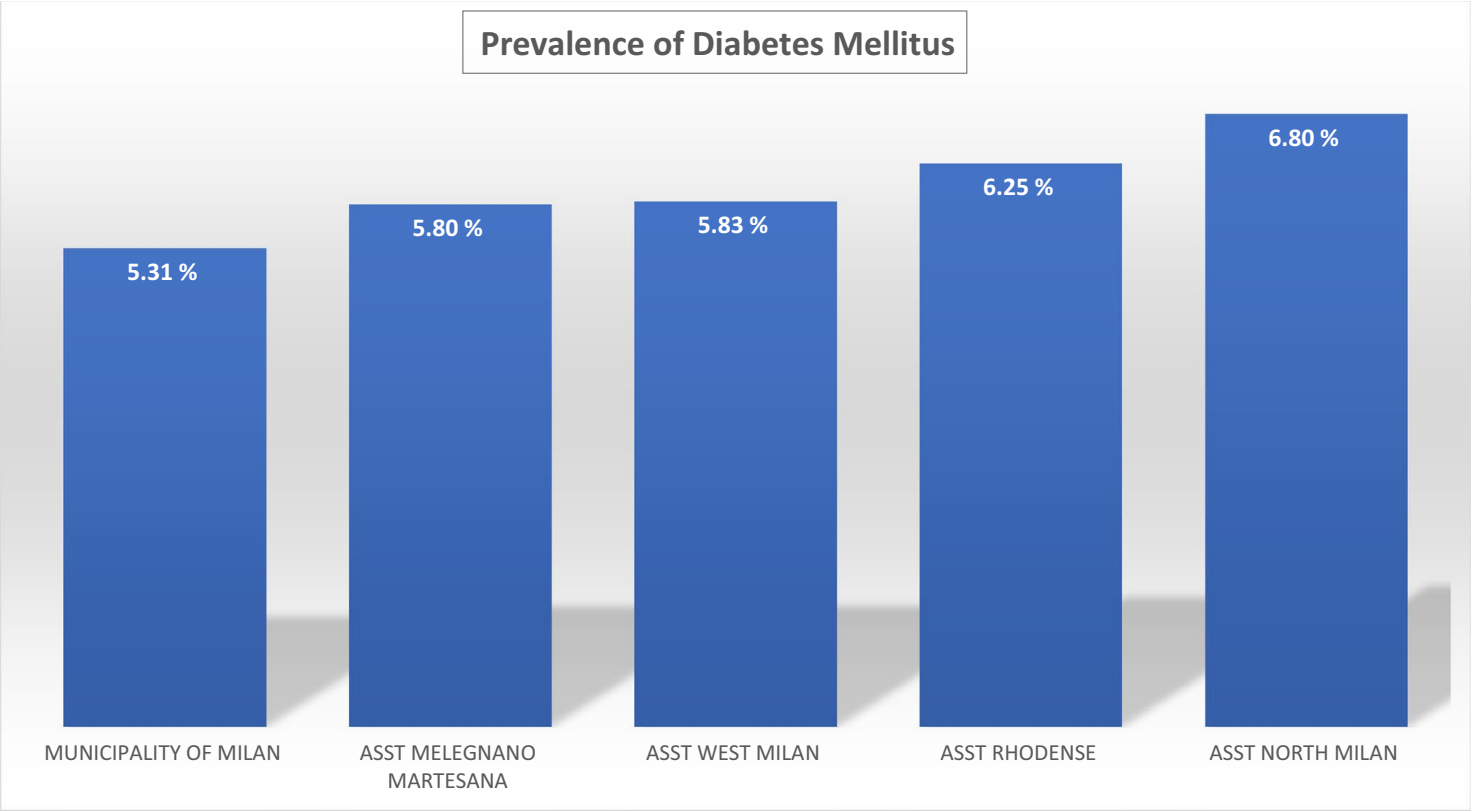
The prevalence of diabetes is lower in downtown Milano and highest in the periphery of Milano Metropolitan Area

**Fig. 2:**
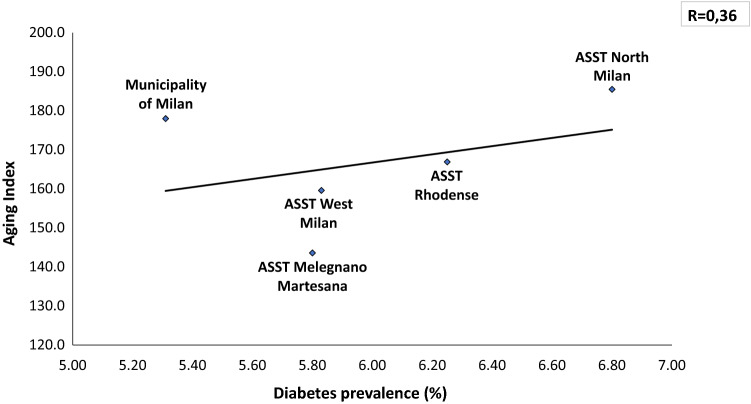
The prevalence of diabetes correlates directly with the Aging index, highest in the periphery of the Milano Metropolitan Area

**Fig. 3:**
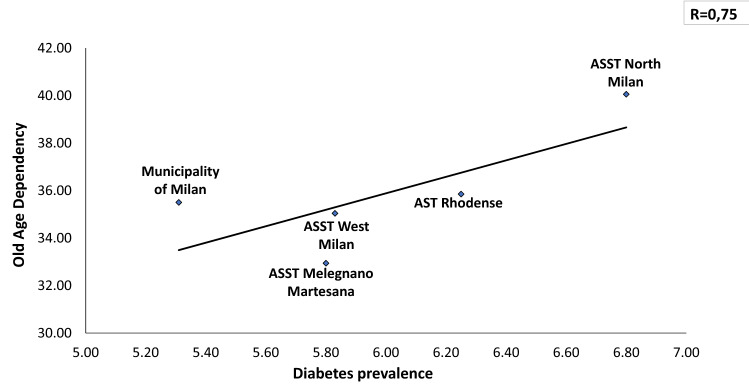
The prevalence of diabetes correlates directly with the Old Age dependency, highest in the periphery of the Milano Metropolitan Area

**Fig. 4:**
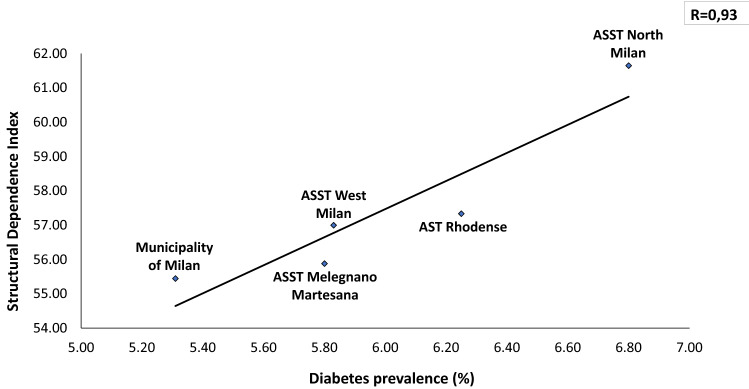
The prevalence of diabetes correlates directly with the Old Age dependency, highest in the periphery of the Milano Metropolitan Area

The average Diabetes Prevalence in the Milano metropolitan Area is 5.74% (males 6.64%, females 4.90%). Seventy-four thousand inhabitants with diagnosis of diabetes mellitus live in downtown Milano, whilst 187.000 patients with diabetes live in the suburbs of Milano. Within the Milano Metropolitan Area diabetes prevalence is strikingly different among downtown (5.31% and peripheral Health Territorial Agencies (e.g., 6.80% diabetes prevalence in ASST North Milano) (Fig. 1). In Fig. 2, the relationship between the Oldness Index and the prevalence of diabetes mellitus in the different ASST of Milano Metropolitan Area is presented. A weak positive correlation is present (*R* = 0.36). Diabetes Prevalence was positively correlated with the Old Age Dependency Index (*R* = 0.75) as well as with the Structural Dependence Index (*R* = 0.93) (Figs. 3 and 4).

**Fig. 5:**
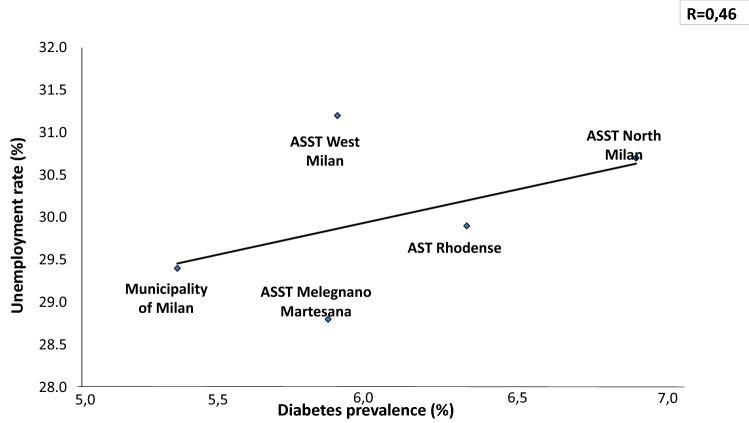
Prevalence of diabetes correlates directly with unemployment rate, highest in the periphery of the Milano Metropolitan Area

**Fig. 6:**
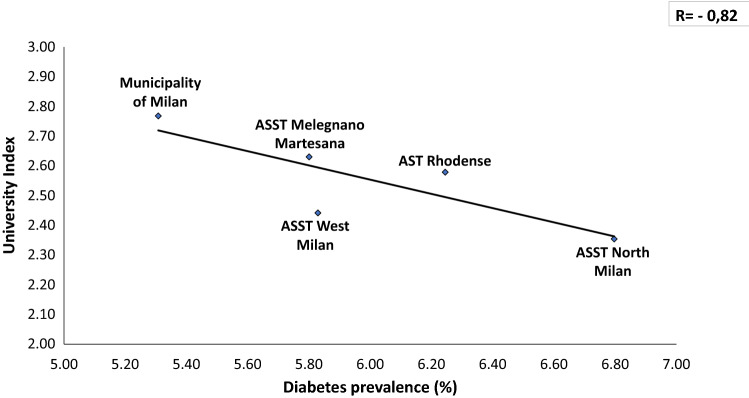
Prevalence of diabetes correlates inversely with the University higher education in the Milano Metropolitan Area

Unemployment rate correlated directly with prevalence of diabetes (*R* = 0.46, Fig. 5), whilst University admission Index was inversely related to it (*R* = 0.82, Fig. 6).

The telemedicine study was conducted in a standard clinical care setting. Following the screening of 65 patients, we included a total of 40 patients, 26 of whom, not utilizing Flash Glucose Monitoring, served as controls. Cases and controls had comparable age (65 years (± 14) vs 68 (± 13) years). At baseline cases had HbA1c 8% (± 1.4), BMI 27 kg/m2 (± 5), 20% [8] of patients were on basal insulin and an oral hypoglycemic agent of those 4 were on basal insulin and a GLP1 RA (mean total daily insulin dose 17 ± 11 IU/Day) and 4 were on basal insulin and SGLT2i (mean total daily insulin dose 18 ± 12 IU/Day), 70% [17] were on basal plus insulin therapy (mean total daily insulin dose 39 ± 24 IU/Day), 0.75% [3] on CSII (Continuous subcutaneous insulin infusion) therapy (mean total daily insulin dose 31 ± 12 IU/Day).

Controls had HbA1c 8% (± 1.8), BMI 30.5 (± 5) kg/m^2^, 69% [18] of patients were on basal insulin and an oral hypoglycemic agent among those 12 were on SGLT2i and basal insulin (mean total daily insulin dose 23 ± 11 IU/Day) and 6 were on GLP1 RA and basal insulin (mean total daily insulin dose 22 ± 11 IU/Day), 31% [8] were on basal plus insulin therapy (mean total daily insulin dose 41 ± 21 IU/Day), none on CSII (Continuous Subcutaneous Insulin Infusion) therapy. Out of 40 patients included 5 were lost at follow-up and 1 patient died. Therefore, out of all controls only 34 completed the follow-up.

According to Ambulatory Glucose Profile (AGP) data obtained by patients FGM data download during three months follow-up (T1), health professionals phoned or mailed patients. Phone calls or e-mails were finalized to titrate insulin dose and improve lifestyle intervention (indoor physical exercise implementation and dietary intervention). After follow-up HbA1c was 7.5 (± 0.9) and BMI was 26.3 kg/m2 (± 5).

Control group at T1 presented HbA1c 8.0% (± 2) and BMI was 31 kg/m2 (± 5).

**Fig. 7:**
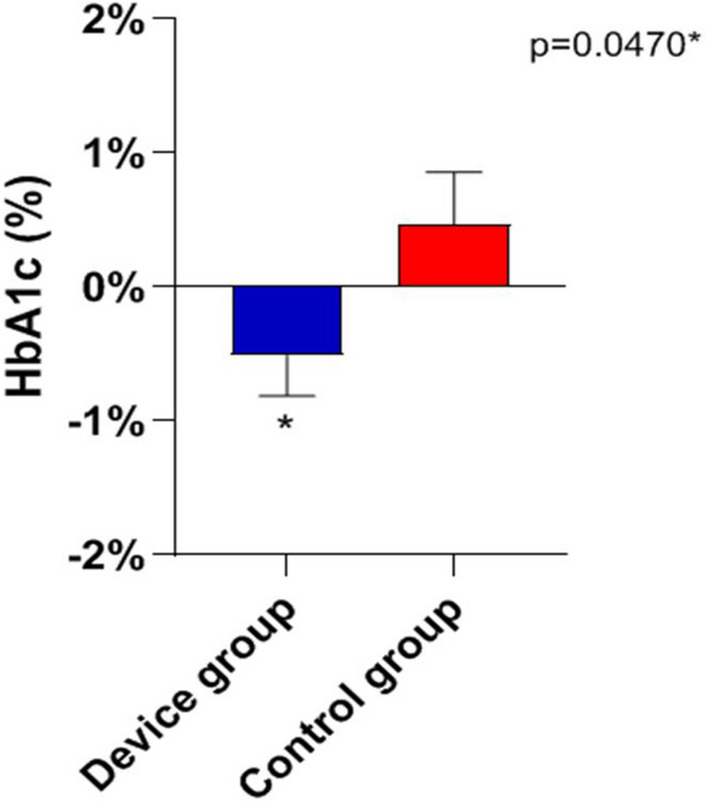
The figure depicts the main results of the pilot study. The group wearing the Telemedicine device demonstrated a significantly lower glycosylated hemoglobin with respect to the control group

Figure 7 depicts the main results of the pilot study. The group wearing the device demonstrated a significantly lower glycosylated hemoglobin with respect to the control group (p < 0.047). No statistical significant different was observed as CV of glycaemia were concerned (Table 1).

**Table 1:** Flash Glucose Monitoring improves glycosylated hemoglobin

	Total	Device	Control	*P*-value (device vs control)
*BMI kg/m*^*2*^				
T0 (mean ± SD)	28.3 ± 5.1	26.7 ± 4.7	30.5 ± 4.9	0.0038*
T1 (mean ± SD)	28.1 ± 5.5	26.1 ± 5.4	30.7 ± 4.6	0.0012*
*BMI kg/m*^*2*^* (T1-T0)*				
Mean ± SD	− 0.1102 ± 1.586	− 0.3719 ± 1.750	0.2164 ± 1.383	0.1740
Mean ± SEM	− 0.1102 ± 0.2065	− 0.3719 ± 0.3093	0.2164 ± 0.2766	
*HbA1c %*				
T0 (mean ± SD)	7.8 ± 1.5	8.0 ± 1.6	7.6 ± 1.3	0.2733
T1 (mean ± SD)	7.8 ± 1.8	7.6 ± 1.4	8.0 ± 2.2	0.3682
*HbA1c % (T1-T0)*				
Mean ± SD	− 0.1531 ± 2.332	− 0.5086 ± 1.785	0.4720 ± 1.928	0.0470*
Mean ± SEM	− 0.1531 ± 0.2915	− 0.5086 ± 0.3018	0.4720 ± 0.3855	

*indicate a* P*-value with a difference between the device and the control groups which is statistically significant

## Discussion

In 1951, Milano Metropolitan Area counted two Million inhabitants. The present population of the Milano Area is 3.227.264 (23% over 65 y) [15]. Starting from 1971 single household increased dramatically from 19.4 to 44.8% of the total [15]. In 2019, 315.000 inhabitants were 65 years old or more [15]. Obesity prevalence in Lombardia region is 7.9% whilst overweight people are 27.5% of the total population. Full sedentariness involve 24% of the resident population with only 35% of inhabitants exercising regularly [19].

Our study confirms the ominous link between urbanization process and diabetes mellitus and obesity in Milano Metropolitan Area [15]. Unfortunately, other chronic-degenerative diseases like hypertension, Chronic Obstructive Pulmonary Disease (COPD) are also more frequent in urban environment rather than in countryside [19]. In the present report, we provide a photograph of the prevalence of diabetes mellitus and obesity in Milano Metropolitan Area in the year 2019. We utilized official data from ISTAT or ATS of Milano Metropolitan Area, data that are publicly available and consultable by general population [14, 15].

Up-to-date, no study has specifically assessed the difference in prevalence of diabetes between the Milano Metropolitan Area and Italian rural areas. Nonetheless, the South of Italy, less urbanized and industrialized than the North of Italy, has an higher prevalence of diabetes mellitus [20]. Interestingly, a similar pattern of high prevalence of diabetes mellitus in rural areas was reported in China [18].

The first photo shoot indicates over 1% difference in diabetes prevalence between Milano downtown (the City) and peripheral ASSTs (the Periphery), with the highest prevalence in the ASST North Milano (Fig. 1). Several factors may explain this difference: a. percent of inhabitants over 65 in different ASST correlates directly with diabetes prevalence in that area (Figs. 2, 3, 4); b. social and economic factors like unemployment rate correlate directly with prevalence of diabetes mellitus (Fig. 5); c. in contrast, level of instruction correlates inversely with percent of affected inhabitants (Fig. 6).

The difference in prevalence between downtown Milano and its peripheries is relevant and deserves several considerations about potential explanations. (1) Genetic/ethnic differences in the resident population should be considered first. Milano has a foreign resident population constituted by 30.9% Asian, European (29,2%), African (21,9%) or South American (18,2%) ancestry. This determines a wider genetic array and therefore different predisposition to diabetes [21]. (2) The Milano Metropolitan Area is located in the middle of the Po Valley, a lowland with a degree of air pollution among the highest ones in Continental Europe. Pollution level differs between downtown Milano and its peripheries. Since air pollution was associated to a higher rate of diabetes and considering that the Northern Areas of Milano are much more industrialized than downtown and Southern Milano areas, this could partially explain the difference in prevalence between ASSTs [22]. (3) An additional environmental factor to explain the difference in diabetes prevalence is external temperature [23]. Global warming has been linked to the world-wide *diabesity* pandemic [24]. Due to different models of urbanization, external temperature may differ up to one Celsius degree in different Metropolitan isles [25]. Populations living in areas exposed to a higher mean temperature may be exposed to higher rates of obesity and diabetes. The principal underlying pathogenic mechanism is a chronic reduction in energy expenditure necessary for thermic regulation processes [26]. (4) Urbanization entails a higher concentration of electrical wiring, wi-fi systems, high-voltage electrical lines causing *e-noise*. All of those generate electromagnetic fields of different intensities and frequencies. Electromagnetic fields are recently suspected of being a co-factor for the increase of diabetes and obesity in the population [27]. High-voltage electrical lines are more abundant in peripheries rather than downtown area in Milano Metropolitan Area. Main high-voltage electric lines are located in the Northern area of Milano [28].

The second photo shoot, taken during the “first wave” of COVID-19 pandemic indicates a higher incidence of SARS-CoV-2 in the Milano metropolitan Area and in the entire Lombardia region, with respect to the rest of Italy: Chance or necessity? In other words, is it possible that the urbanization per se had caused a higher incidence and worse prognosis of COVID-19? What is the reason of the highest mortality around the world seen in Lombardia region? In large cities, like Milano, population density is higher making difficult social distancing [29]. The second factor conceivably linking urbanization, diabetes and COVID-19 is the oldness of population. Our mapping of the Milano Metropolitan Area clearly shows a positive correlation between 3 indexes involving aging of the population and prevalence of diabetes in different ASSTs (Figs. 2, 3, 4). It is known that older population has the highest mortality rate in all coronavirus and influenza virus outbreaks [9, 17, 30]. Approximately 85% of diabetic patients are obese. In fact, the concomitance of diabetes and aging presumably constitute a synergistic negative factor for the COVID-19 outcome. All authors agree on the fact that diabetic patients have a worse prognosis than non-diabetic ones [17, 31, 32]. The clinical picture of COVID-19 is even worse when obesity is associated with diabetes [9, 33]. Hypertension, cardiovascular disease and COPD have been also called responsible for a most serious prognosis of SARS-CoV2 infection [30, 34]. Therefore, urban diabetes and urban obesity can be suggested as a facilitating factor to reach the high incidence of COVID-19 in Metropolitan Areas worldwide (Milano, Paris, London, New York City, San Paulo, Santiago, Bogota). To note that the 3 months lock-down have determined an increase in obesity prevalence in several countries [35]. Viceversa, adherence to a strict nutritional management and follow-up reduces successfully body weight [36].

Unemployment rate was directly correlated to diabetes prevalence in the Milano metropolitan Area. Intriguingly, unemployment also correlates to susceptibility and mortality in previous influenza epidemics [16, 37]. Conversely, COVID-19 pandemic is causing an increase in suicides among unemployed people [38]. Also low-education level is associated to both a higher prevalence of obesity and diabetes and a lower prognosis of COVID-19 [39, 40].

What are the lessons the health System should learn after COVID-19 outbreak in Milano Metropolitan Area to be put in place during the “second wave “of epidemic? First, diabetes management needs to be optimal even during social distancing. This is to reduce the impact of having a poorly controlled diabetic population during the ongoing”second wave” wave of COVID-19. Secondly, air pollution must be reduced, since it may facilitate the viral diffusion. Finally, territorial medicine should be implemented to prevent, or treat in its initial phase, COVID-19.

The pilot study we presented herein was conducted during the “first wave” of epidemic and works as a “proof of concept” indicating that remote glucose control allows better glycemic control in a condition of social distancing as the lock-down occurred in March and April 2020. Previous studies had shown the possibility of optimizing glucose control via Flash Glucose Monitoring [41, 42], but, at our knowledge, this is the first study utilizing the system during a lock-down (Fig. 7) to bend the “second wave” of contagion.

In conclusion, in January 2020, the casual (*the Chance*) concomitance of three factors—Urbanization-derived population density/air pollution, diabetes – determined the conditions for an unavoidable (*the Necessity*, [43]) perfect storm in presence of SARS-CoV2 diffusion. We reported epidemiologic and sociodemographic data in the Milano Metropolitan Area with a picture of the diabetic population taken at the beginning of the SARS-Cov-2 pandemic diffusion. We speculate that the environmental and health conditions of Milano may have favored the high diffusion and the high severity of COVID-19. Population density, pollution, diabetes prevalence, aging index may constitute some clues that need to be further investigated to better understand the high diffusion and mortality rate of COVID-19 in the Milano Metropolitan Area and Lombardia region. We also presented original data showing the efficacy of a Telemedicine Aid during a lock-down period, suggesting that Telemedicine should become more and more a methodology for emergency periods.
